# Supplementary material for: Impacting Fuelling Dynamics Towards Tunable Liquid Metal Machine
Source: Exploration (Beijing). 2026 Oct 6;6(5):70227. doi: 10.1002/exp2.70227 (PMC13643008; doi:10.1002/exp2.70227)
Supplement: Supplementary file 1 [file EXP2-6-70227-s002.docx]

**Impacting Fueling Dynamics towards Tunable Liquid Metal Machine**

**This Word File includes:**

1. Supplemental Figures 1-5
2. Supplemental Note 1: Migration mechanism of the fuel region
3. Supplemental Note 2: Stabilization mechanism of the fuel region
4. Supplemental Note 3: Force Analysis and Dynamics Characterization
5. Supplemental Note 4: Comprehensive behavioral analysis of LMM under different confining obstacle materials
6. Supplemental Note 5: Enhanced heat transfer mechanism of LMM dynamic behavior

**Other supplemental information for this manuscript includes the following:**

Supplemental Video 1: Beginning of liquid metal machine activation process - wetting and floating of aluminum fragments (volume: 200 μL, operation time: 1 min)

Supplemental Video 2: Completion of liquid metal machine activation process - fuel region transfer to the tail (volume: 200 μL, operation time: 10 min)

Supplemental Video 3: Impact and rebound of liquid metal machine in two-dimensional plane (volume: 100 μL, operation time: 50 min)

Supplemental Video 4: Reciprocating motion of liquid metal machines in the one-dimensional channel with quartz obstacles (volume: 100 μL, operation time: 60 min)

**Supplemental Video 5:** ∞-shaped fuel region trajectory at the tail of the liquid metal machine during one-dimensional motion (volume: 100 μL, operation time: 75 min)

Supplemental Video 6: Early-stage end impact-turning dynamics with quartz obstacles (volume: 100 μL, operation time: 60 min)

Supplemental Video 7: Late-stage end impact-turning dynamics with quartz obstacles (volume: 100 μL, operation time: 80 min)

Supplemental Video 8: Dye-traced fluid flow visualization during end impact-turning with quartz obstacles (volume: 100 μL, operation time: 80 min)

Supplemental Video 9: End impact-turning dynamics with platinum obstacles (side perspective, volume: 100 μL, observation time: 60 min)

Supplemental Video 10: End impact-turning dynamics with platinum obstacles (top perspective, volume: 100 μL, observation time: 80 min)

Supplemental Video 11: Early-stage end impact-turning dynamics with graphite obstacles (volume: 100 μL, operation time: 60 min)

Supplemental Video 12: Late-stage end impact-turning dynamics with graphite obstacles (volume: 100 μL, operation time: 80 min)

**Supplemental Video 13:** Heat transfer demonstration of liquid metal machine operation (volume: 100 μL, operation time: 60 min)

**Supplemental Figure 1.** SEM images and corresponding EDS elemental distribution maps of the fluid part of LMM at 3 h after activation.

**Supplemental Figure 2.** SEM images and corresponding EDS elemental distribution maps of the moving fuel region’s upper part.

**Supplemental Figure 3.** SEM images and corresponding EDS elemental distribution maps of the moving fuel region’s lower part.

**Supplemental Figure 4.** **Top views of LMM achieving the directional change process** **in one-dimensional systems with** **platinum obstacles (*V*_LMM_= 100 μL).** a. The process of LMM impacting the platinum obstacle and then turning at the early stage; b. The process of LMM impacting the platinum obstacle and then turning at the late stage.

**Supplemental Note 1: Migration mechanism of the fuel region**

We investigate the fundamental mechanisms governing the directional migration of aluminum fragments from their initial distribution to the tail region of self-propelled LMM. This migration process is essential for establishing stable propulsion and involves complex interactions between wetting phenomena, electrochemical reactions, and fluid dynamics.

The initial contact between GaIn_10_ and aluminum triggers a sequence of well-documented metallurgical phenomena (**Figure 1b**). ^[1]^ Firstly, for the Ga-Al system, the corrosion effect operates through several mechanisms, manifesting as the reduction of solid surface energy through LM adsorption, and leads to enhanced deformation and crack propagation. The native aluminum oxide layer is penetrated by gallium through the formation of Ga-Al intermetallic compounds. Gallium preferentially diffuses along aluminum grain boundaries, and at the Ga-Al interface, intermetallic compounds $\mathrm{Ga}_{x}\mathrm{Al}_{1-x}$ rapidly form, creating brittle interfacial layers that promote mechanical failure of aluminum, causing fragmentation. ^[2-4]^ When the oxide layer is disrupted, the system spontaneously forms an electrochemical battery in the alkaline solution environment: ^[5]^

Anode:

$Al+4\mathrm{OH}^{-}\longrightarrow{\mathrm{Al}\left( \mathrm{OH} \right)}_{4}^{-}+3e^{-}$	(1)

Cathode:

$2H_{2}O+2e^{-}\longrightarrow H_{2}+2\mathrm{OH}^{-}$	(2)

Overall:

$2Al+6H_{2}O\longrightarrow{2\mathrm{Al}^{3+}+ 3H}_{2}+6\mathrm{OH}^{-}$	(3)

Under alkaline solution conditions, the battery potential provides sufficient driving force for continuous reactions. In addition, experimental observation shows that aluminum fragments consistently appear at the LMM’s surface rather than remaining dispersed throughout the droplet volume (**Figure 2b**), This phenomenon may be attributed to the density difference between GaIn_10_ and aluminum, which gives rise to a buoyancy force acting on aluminum fragments. For a circular fragment with radius *R* and thickness *δ*, the buoyancy force can be expressed as:

$F_{b}=\pi R^{2}g\delta\left( \rho_{\mathrm{Ga}\mathrm{In}_{10}}-\rho_{\mathrm{Al}} \right)$	(4)

After aluminum fragments dissolve at the droplet surface, concentration gradients of reaction products develop, resulting in surface tension variations. These gradients induce Marangoni flows directed from regions of low surface tension (high aluminum coverage) to regions of high surface tension (low aluminum coverage), and providing the primary driving force for droplet motion.

Driven by the Marangoni effect described above, the motion through the aqueous medium generates a characteristic flow field that transports surface particles toward the LMM’s rear. As shown in **Figure 3e**, fluid at the LMM’s front flows backward along the surface, generating rearward shear stress. This stress peaks near the droplet equator, applying a strong force that pushes aluminum fragments rearward. In contrast, a recirculation zone forms in the LMM’s rear region, where shear stress reaches minimum. Such a non-uniform shear stress distribution applies directional forces that continuously drive aluminum fragments toward the tail, maintaining the asymmetric fuel distribution essential for stable propulsion. The coupling between these flow systems ensures a stable fuel region of aluminum fragments at the tail. ^[6]^

**Supplemental Note 2: Stabilization mechanism of the fuel region**

The fuel region consistently maintains its position at the tail of LMM throughout the operational lifetime (**Figure 2b**). At the onset of the process, this stabilization is governed by a combination of hydrodynamic trapping, electrochemical feedback, and mechanical anchoring. As the system evolves, and the fuel region undergoes continuous changes in both area and composition, these mechanisms remain active and dynamically adjust to sustain its position and stability at the droplet tail.

In the early stage of LMMs’ motion, the stability of small fuel regions, typically occupying less than 1% of the LMM surface area, is achieved through coordinated hydrodynamic, electrochemical, and mechanical interactions. The moving droplet generates a characteristic wake containing recirculating flow patterns (**Figure 3e**). Within this wake, vortices create enclosed or slowly evolving streamlines that effectively trap aluminum fragments. Once particles are drawn into this zone, they become entrained in the recirculating flow and remain captured by the vortex structure. ^[7]^ Unlike the front and side regions of the droplet, the rear wake area lacks such strong vortical activity, forming a relatively quiescent zone. This reduced shear stress environment is further stabilized by the characteristic pressure distribution of the wake structure, which creates a low-pressure region at the droplet tail that opposes particle escape through pressure-driven retention. ^[6, 8]^

The electrochemical reaction occurring at the tail of the LMM plays a pivotal role in maintaining positional stability by establishing a self-correcting feedback mechanism. Hydrogen bubbles generated at fuel region are expelled in the forward motion, producing thrust that aligns with the droplet's direction. This directional match not only contributes to propulsion but also enhances positional stability. If the fuel region were displaced from its tail position, the misaligned hydrogen release results in asymmetric thrust, thereby inducing restoring forces that drive the pool back toward the equilibrium location.

Simultaneously, aluminum fragments develop strong adhesive interactions with the LMM surface through intermetallic bond formation at the Ga-Al interface, while the rapid formation of Al(OH)_3_ creates a gel-like matrix that enhances surface adhesion and provides mechanical stability. (**Figure S3**). ^[9-12]^

The stable fuel region localization results from dynamic equilibrium between competing forces. Hydrodynamic drag directs the pool toward the tail region, where wake structures provide positional bias. Electrochemical forces from hydrogen bubble momentum transfer contribute both propulsive and stabilizing effects. Adhesive forces resist displacement through Ga-Al bonding, while geometric interlocking between irregular aluminum fragments reinforces cluster stability. This force balance explains the natural migration and retention of fuel regions in the wake region, where reduced hydrodynamic stress, surface energy minimization, and electrochemically-driven positional feedback sustain localization throughout droplet motion.

As the fuel region area and composition evolve through reaction product accumulation, the fundamental stabilization mechanisms undergo adaptive adjustments to maintain positioning at the LMM tail. The accumulated aluminum hydroxide and other reaction products integrate structurally with aluminum fragments through mechanical embedding and chemical bonding, forming a cohesive composite unit. This integration transforms the discrete particle system into a unified entity that responds collectively to external disturbances, with perturbation energy distributed throughout the structure rather than causing individual fragment dispersion.

Concurrent with compositional changes, the electrochemically active region shifts spatially to follow aluminum fragment redistribution, ensuring continuous hydrogen generation and sustained propulsive forces during motion. This adaptive repositioning preserves directional stability despite ongoing structural evolution. As the fuel region continues expanding, it eventually extends beyond the primary wake region and becomes entrained within bilateral vortex zones at the LMM rear, resulting in the characteristic ∞-shaped trajectory patterns observed in late-stage (**Figure 2d**). ^[13]^

In summary, the stable operation mechanism of the LMM operates continuously across different stages, from initial activation through overall compositional evolution, endowing the LMM with anti-disturbance capabilities and adaptive functionality.

**Supplemental Note 3: Force Analysis and Dynamics Characterization**

**1. System Characterization**

The LMM consists of eutectic gallium-indium alloy (GaIn_10_: 90 wt% Ga, 10 wt% In) mixed with aluminum particles at a mass ratio of 100:1.08. For the droplet volume of 100 μL used throughout these experiments, the total mass is approximately 0.618 g. Since aluminum represents only 1.07% of the initial mass and undergoes continuous consumption through the electrochemical reaction (**Eq. S3**), we approximate the droplet mass using the GaIn_10_ component alone for force calculations.

The physical properties of GaIn_10_ at room temperature (25 ℃) have been characterized in previous studies, ^[14]^ with density ρ = 6250 kg·m^-3^, surface tension γ = 0.718 N·m^-1^, and dynamic viscosity μ = 2.1 mPa·s. ^[15]^ The droplet adopts an ellipsoidal geometry when confined in the 6 mm diameter channel, with measured dimensions of 6.0 mm (width), 4.29 mm (height), and 6.27 mm (length), yielding total surface area approximately 125 mm^2^ and cross-sectional area approximately 20.2 mm^2^.

To characterize stage-dependent force dynamics, we examine the system at 60 and 80 minutes as representative early and late-stage benchmarks, respectively. Fuel region coverage increases from 1.71% at 60 minutes (absolute area ≈ 2.1 mm^2^) to 8.74% at 80 minutes (absolute area ≈ 10.9 mm^2^). Kinematic data from high-speed imaging (400 fps) were analyzed over three consecutive motion cycles at each time point, yielding mean velocities of 42.0 ± 0.3 mm·s⁻¹ and 25.0 ± 0.3 mm·s⁻¹ at 60 and 80 minutes respectively. Peak accelerations during collision were identified from velocity-time profiles using central difference methods with appropriate smoothing. Forces were subsequently estimated using Newton's second law combined with established fluid mechanics frameworks.

**2. Directional Considerations for Electrochemical Propulsion**

The electrochemical propulsion force arises from Marangoni flow driven by surface tension gradients at the fuel region boundaries. Since the fuel region is distributed on the curved droplet surface (concentrated in the trailing region), the propulsive force direction is perpendicular to the local surface where active aluminum fragments generate the surface tension reduction. This geometric relationship has important implications for the effective propulsion along the motion direction.

In the quantitative analysis presented in this Supporting Information, we consider the effective propulsion along the motion direction, which can be expressed as:

$F_{e, eff}= F_{e}\times cos\theta$	(5)

where θ is the angle between the local surface normal at the active reaction sites and the horizontal motion direction. At 60 minutes, with the fuel region occupying only 1.71% of surface area and active aluminum concentrated at the upper edge of the compact tail region, the angle *θ* is small. Thus, the effective horizontal propulsion is very close to the total propulsive force magnitude, and our horizontal force balance analysis is directly applicable with minimal correction.

At 80 minutes, fuel region expansion to 8.74% coverage leads to a larger perimeter with active aluminum fragments distributed along the upper edge. As described in the main text, reaction product accumulation at the bottom of the fuel region causes the thrust point to migrate upward. During collision-rebound cycles, this upward shift affects the horizontal component of propulsion. When portions of the active aluminum distribution extend to upper surface locations on the curved tail, the angle *θ* may increase to 30 to 45 degrees, yielding cos*θ* in the range 0.71 to 0.87. This geometric effect would reduce the effective horizontal propulsion compared to the total propulsive force magnitude.

However, since our force estimates are derived from measured velocities and force balance requirements during steady horizontal motion, the values of *F*_e_ reported throughout this analysis already represent the effective horizontal component. The force balance equations use *F*_e_ to denote this effective horizontal propulsion, recognizing that the total three-dimensional propulsive force magnitude may be somewhat higher depending on the spatial distribution of active aluminum fragments along the fuel region perimeter. This directional consideration contributes to the reduced propulsive efficiency observed in the late stage, in addition to the dilution of active aluminum over a larger perimeter and the weakening of directional asymmetry.

**3. Force Analysis of Early-stage**

**3.1. Uniform Motion Phase of Early-stage**

During steady motion in the channel center, the LMM maintains approximately constant velocity with near-zero acceleration. The propulsive forces must balance resistive forces according to:

$F_{e}+ F_{h}= F_{v}+ F_{f}$	(6)

where $F_{e}$ is electrochemical propulsion pointing in the motion direction, $F_{h}$ is hydrogen bubble thrust, $F_{v}$ is viscous drag, and $F_{f}$ is contact line friction.

The electrochemical propulsion arises from Marangoni flow driven by surface tension gradients at the fuel region boundaries. At 60 minutes, the fuel region occupies only 1.71% of the surface, concentrated at the droplet tail. This compact distribution creates strong directional asymmetry in the chemical environment. Previous studies have shown that electrochemical reactions at liquid metal surfaces generate substantial surface tension gradients through local pH changes and electrochemical potential variations. ^[16-18]^

Regarding hydrogen bubble thrust, theoretical considerations suggest that momentum transfer from bubbles departing the droplet surface is orders of magnitude smaller than electrochemical propulsion. ^[18]^ While bubbles are clearly visible in videos and indicated in **Figure 3b** with notation ($F_{e}+ F_{h}$), the practical contribution is negligible, contributing less than 0.1% of total propulsion. We retain this notation for completeness but $F_{h}$ can be safely neglected in quantitative calculations.

Viscous drag on the moving droplet arises from flow of the surrounding 0.5 mol·L^-1^ NaOH solution around the droplet. And it can be estimated as：

$F_{v} = \frac{1}{2}\rho_{s}v^{2}C_{d}A$	(7)

where A is the elliptical cross-sectional area, $C_{d}$ is the drag coefficient, and $\rho_{s}$≈ 1020 kg·m^-3^ is the density of 0.5 mol·L^-1^ NaOH solution.

The Reynolds number characterizes the flow regime:

$Re = \frac{\rho_{s}vL}{\mu_{s}}$	(8)

where $\mu_{s}$ ≈ 1.1 mPa·s is the dynamic viscosity of the NaOH solution. At 60 minutes, this yields Re ≈ 244, indicating transitional flow where both inertial and viscous effects contribute. Using $C_{d}$≈ 0.8 for this regime yields F_v_ ≈ 4.3×10^-5^ N.

Contact line friction arises from contact angle hysteresis at the three-phase boundary. Under continuous motion with thin film lubrication, the effective hysteresis is substantially reduced from static values. From the force balance requirement, $F_{f}$ ≈ 4.0×10^-5^ N at 60 minutes, giving the sum $F_{v}+ F_{f}$ ≈ 8.3×10^-5^ N. Therefore, Fe ≈ 8.3×10^-5^ N.

And the kinetic energy:

$E_{k}=\frac{1}{2}mv^{2}$	(9)

At this stage, $E_{k}$ ≈ 5.4×10^-7^ J.

**3.2. Collision and Deceleration Phase of Early-stage**

Upon contact with the end obstacle, the LMM enters a complex collision process involving deceleration, maximum compression, and rebound phases. During the deceleration phase, the LMM continues moving forward while experiencing increasing deformation. All forces present during uniform motion persist, with two additional forces: lateral support force $F_{s}$ from the obstacle and capillary restoring force $F_{c}$ from the deformed interface resisting further compression. The force balance becomes:

$F_{e}+ F_{h}-F_{v}-F_{f}-F_{s}-F_{c}= ma$	(10)

where a < 0. As deformation increases, $F_{c}$ builds up from zero at initial contact. The measured peak deceleration is a = -138 ± 3 mm·s^-2^, yielding ma = -8.5×10^-5^ N. This represents the maximum measurable deceleration during the compression phase, though not necessarily at the instant of maximum compression where velocity reaches zero. At the peak deceleration point, the combined resistance is:

$F_{s}+ F_{c}=F_{e}+F_{h}-F_{v}-F_{f}- ma\approx1.65\times{10}^{-4} N$	(11)

At maximum compression, velocity reaches zero momentarily while the droplet transitions from forward to reverse motion. At this instant, both $F_{s}$ and $F_{c}$ reach their maximum values, storing elastic energy approximately equal to the initial kinetic energy (accounting for viscous dissipation). Since v = 0, both $F_{v}$ and $F_{f}$ vanish. The force balance becomes:

$F_{s}+ F_{c}-F_{e}-F_{h}= ma$	(12)

where a now points in the reverse direction. The acceleration magnitude at this instant is theoretically maximum, but measurement uncertainty is high due to the brief duration and numerical differentiation instability near v = 0. We therefore use the measured peak values during compression and rebound phases for quantitative force estimates.

**3.3. Rebound and Acceleration Phase of Early-stage**

After maximum compression, the stored elastic energy drives rebound. The droplet accelerates in the reverse direction while remaining deformed. Both Fs and $F_{c}$ act in the current motion direction that away from obstacle, while Fe still points in the original direction since the fuel region has not yet transferred. As velocity increases, Fv and Ff oppose the current motion. The force balance is:

$F_{s}+F_{c}-F_{e}-F_{h}-F_{v}-F_{f}= ma$	(13)

where a > 0 in the current motion direction. The measured peak rebound acceleration reaches a_max_= 285 mm·s^-2^, yielding ma= 1.76×10^-4^ N. At this point, $F_{s}+F_{c}\approx3.51\times{10}^{-4} N$. This substantial force, approximately four times the electrochemical propulsion, drives the rapid rebound characteristic of early-stage collisions. The high elastic force reflects the large deformation and high surface tension of liquid metal (γ = 0.718 N·m^-1^).

**3.4. Fuel Region Transfer and Motion Resumption of Early-stage**

As the LMM continues rebounding, internal Marangoni flow causes the fuel region to migrate from the tail toward the leading edge. The deformation gradually decreases, reducing both $F_{s}$ and $F_{c}$. When the fuel region reaches the bottom of the LMM that perpendicular to motion direction, the horizontal component of $F_{e}$ becomes zero. At this critical point, acceleration reaches zero and the force balance is:

$F_{s}+F_{c}=F_{v}+F_{f}$	(14)

where all forces are horizontal components. The remaining elastic forces balance the resistive forces as the LMM transitions from accelerating to uniform motion.

Subsequently, the fuel region continues migrating to the new tail position, re-establishing $F_{e}$ in the new motion direction. The lateral support $F_{s}$ vanishes as contact is lost, and $F_{c}$ diminishes to zero as the LMM returns to equilibrium shape. The force balance returns to Eq. S5 with $F_{e}$ now pointing in the reversed direction. At 60 minutes, this entire sequence completes in less than 1 second, enabling efficient directional reversal.

The collision process involves conversion of kinetic energy (5.4×10^-7^ J) into surface deformation energy and viscous dissipation. The surface deformation energy can be approximated as γ·ΔA, where ΔA is the excess surface area created during compression. During rebound, a fraction of this stored energy is recovered as kinetic energy in the reverse direction. The rebound velocity, approximately 38 mm·s^-1^ based on subsequent motion, corresponds to kinetic energy of approximately 4.5×10^-7^ J, yielding an effective rebound coefficient of approximately 0.9. This high recovery efficiency reflects the dominance of elastic capillary forces over viscous dissipation at this stage.

**4. Force Analysis of Late-stage**

**4.1. Uniform Motion Phase of Late-stage**

The force balance equation remains unchanged (Eq. S5), but individual force magnitudes shift due to reduced velocity (v = 25.0 mm·s^-1^) and altered fuel region structure. The Reynolds number decreases to Re ≈ 145. Using Cd ≈ 0.9 for this lower Re, viscous drag decreases to $F_{v}$ ≈ 2.3×10^-5^ N, scaling roughly with v^2^ as expected in the transitional regime.

Contact line friction increases to $F_{f}$ ≈ 4.8×10^-5^ N due to aluminum hydroxide deposition. From force balance, $F_{e}$ ≈ 7.1×10^-5^ N, representing a 14% decrease despite the 5-fold larger fuel region area.

This degradation reflects systematic changes in fuel region structure. The bottom portion accumulates passive reaction products (intermetallic phases and aluminum hydroxide precipitates) while active aluminum fragments migrate to the upper edge and disperse along the expanded perimeter. This dilution reduces the effective surface tension gradient. Additionally, the distributed active sites generate propulsive forces from multiple locations with varying orientations, weakening the net forward thrust. As discussed in Section 2, the upward migration of thrust points at 80 minutes further reduces the horizontal propulsion component through geometric effects. The kinetic energy decreases to $E_{k}\approx1.9\times{10}^{-7}$ J, a 65% reduction from 60 minutes.

**4.2. Collision Dynamics of Late-stage**

The collision sequence follows the same phases as 60 minutes but with reduced force magnitudes reflecting the lower kinetic energy. Peak deceleration is a = -85 ± 3 mm·s^-2^, and peak rebound acceleration is a = 172 ± 4 mm·s^-2^, yielding estimated peak forces $F_{s}+ F_{c}\approx1.0\times{10}^{-4} N$ during deceleration and ${F_{s}+F}_{c}\approx2.22\times{10}^{-4} N$ during rebound.

The reduced elastic forces reflect the lower kinetic energy and smaller deformation extent. The rebound velocity (approximately 22 mm·s^-1^) corresponds to kinetic energy of approximately 1.5×10^-7^ J, yielding a rebound coefficient of approximately 0.89, similar to 60 minutes. The collision mechanics remain fundamentally unchanged; only the force magnitudes scale with the available kinetic energy.

**4.3. Multi-Impact Behavior and Fuel Region Transfer of Late-stage**

The critical difference at 80 minutes is the insufficient kinetic energy for single-impact fuel region transfer. The reduced internal Marangoni flow velocity, caused by the degraded surface tension gradients and dispersed active aluminum distribution, slows fuel region migration. Additionally, enhanced aluminum hydroxide adhesion at the original location resists detachment.

Consequently, after the first rebound, the fuel region does not complete transfer through the bottom position. Instead, it remains partially at the original location, and $F_{e}$ continues generating components that drive the droplet back toward the obstacle. The droplet undergoes multiple oscillatory impacts, typically 3-5 cycles over 5-10 seconds, before the fuel region completes transfer.

During these oscillations, each impact provides additional perturbation to the fuel region. The cumulative effect of multiple gentle collisions eventually overcomes the adhesion forces and completes the migration that a single strong collision would have achieved at 60 minutes. This multi-impact behavior characterizes the late-stage inefficient turning dynamics and directly explains the increased turning time observed in **Figure 2d**.

**5. Dimensionless Characterization**

Several dimensionless numbers help characterize the physical regime governing the LMM dynamics. The capillary number quantifies the ratio of viscous to surface tension forces:

$Ca = \frac{\mu_{s}v}{\gamma}$	(15)

At 60 minutes: Ca ≈ 6.4$\times{10}^{-5}$, while at 80 minutes, Ca ≈ 3.8$\times{10}^{-5}$.

Both values are much less than unity, confirming that surface tension forces dominate over viscous effects throughout the operational lifetime.

The Bond number compares gravitational to surface tension forces, using the density difference between liquid metal and surrounding solution:

$Bo = \frac{\Delta\rho gL^{2}}{\gamma}$	(16)

Under the current experimental conditions, Bo ≈ 2.79$\times{10}^{-3}$, this value much less than unity indicates that gravitational effects are negligible, and the motion is governed by interfacial forces rather than buoyancy or sedimentation.

The Reynolds number characterizes the flow regime in the surrounding fluid:

$Re=\frac{\rho_{s}vL}{\mu_{s}}$	(17)

At 60 minutes, Re ≈244, and at 80 minutes, Re ≈145.

Both values place the system in a transitional flow regime, intermediate between purely viscous (Stokes, Re << 1) and fully inertial (Re >> 1) limits. In this regime, both viscous dissipation and inertial effects contribute significantly to the drag force. This justifies the use of empirical drag coefficient correlations that account for both mechanisms, rather than purely analytical Stokes drag formulas that would underestimate resistance in this Reynolds number range.

The Weber number compares inertial to surface tension forces:

$We =\frac{\rho_{s}v^{2}L}{\gamma}$	(18)

At 60 minutes, We ≈ 1.57$\times{10}^{-2}$, at 80 minutes, We ≈ 5.57$\times{10}^{-3}$. These values much less than unity confirm that droplet deformation during collision is governed primarily by capillary forces rather than inertia.

The dimensionless analysis provides a consistent physical picture across all parameters. Throughout the operational lifetime from 60 to 80 minutes, the system remains firmly in a surface-tension-dominated regime (Ca, Bo, We all << 1) with transitional flow conditions in the surrounding fluid (Re ~ 145-244). The decrease in all velocity-dependent dimensionless numbers (Ca, Re, We) reflects the overall slowing of motion as propulsive efficiency degrades, but the fundamental physical regime remains unchanged. Surface tension continues to dominate both the propulsion mechanism through Marangoni gradients and the collision dynamics through capillary restoration, while viscous and inertial effects remain secondary but non-negligible contributors to the overall force balance.

**Supplemental Note 4:** **Comprehensive behavioral analysis of LMM under different confining obstacle materials**

1. **Introduction**

This section provides detailed analysis of the physical and electrochemical interactions involved in the behavior of LMMs within motion systems featuring different confining obstacle materials. Based on the research findings mentioned in the manuscript, we conduct an in-depth exploration of the material-dependent mechanisms underlying the observed differences in motion period stability, velocity characteristics, turning time percentages, and electrochemical responses.

2. **Material properties and interface characteristics**

The three obstacle materials investigated in this paper each possess unique physical and chemical properties, representing non-metallic insulator, non-metallic conductor, and metallic conductor, respectively. These characteristics fundamentally influence their interactions with LMMs.

Quartz exhibits stable physical and chemical properties with a relatively smooth surface, providing a relatively uniform collision interface. Its moderate and uniform elastic modulus (~75 GPa) and non-participation in electrochemical reactions ensure that surface energy (~73 mJ∙m^-2^) and the efficiency of collision energy conversion to rebound energy remain relatively constant over extended periods, providing a stable impact-turning environment. ^[19, 20]^ Platinum possesses high electrical conductivity (10^5^-10^6^ S∙m^-1^), high surface energy (~2000 mJ∙m^-2^), and excellent electrocatalytic activity, particularly prominent in water decomposition reactions. ^[21]^ Graphite features a layered structure with microscopic surface inhomogeneity, moderate anisotropic electrical conductivity (10^3^-10^5^ S∙m^-1^), surface energy (~70-80 mJ∙m^-2^), and interlayer structure connected by weak van der Waals forces. ^[22]^ Its π electron system confers strong adsorption capability for various substances, while structural anisotropy leads to complex mechanical and electrochemical behaviors.

3. **Comprehensive experimental observations**

We summarize the key experimental observations for LMMs operating with different obstacle materials:

**Table S1. Effect of interfacial interaction on LMM reciprocating characteristics**

| **Material** | **Quartz** | | **Platinum** | | **Graphite** | |
| --- | --- | --- | --- | --- | --- | --- |
| **Time stage** | **Early** | **Late** | **Early** | **Late** | **Early** | **Late** |
| **Peak voltage** | No | | Negative:  -2 mV; Positive:  0.5 mV | Negative: -0.015mV; Positive: 0.005mV | Positive：  >60mV; Stable：<10mV | Contact:  -2mV;  Non-contact: 1mV |
| **Turning time percentage** | ~2.9% | Progressively increase | ~2.7% | Progressively decrease | ~30% | Stably decrease |
| **Period** | Most consistently stable | | Stable | Step-like change occurs in the final stage | Long, significant fluctuation | Long, reduced fluctuation |
| **Velocity** | Remains relatively stable, gradual decrease | | Stable | Rapidly decreases | Relatively stable | Relatively stable |
| **Fuel region location** | Bottom | | Upper surface slides to the opposite side (during turning) | | Bottom | |

Based on the distinct impact and turning dynamics observed compared to quartz obstacle systems, it can be concluded that in platinum or graphite obstacle systems, the behavior patterns can no longer be explained by simple mechanical interactions, thus necessitating a more in-depth analysis of the coupled physicochemical processes.

**4. Mechanistic analysis**

**4.1 Quartz obstacle system: Pure mechanical effect control group**

In this system, LMMs exhibit the most consistent motion period and moderately stable velocity. The driving mechanism is primarily attributed to the intrinsic Ga/In-Al galvanic cell system of the LMM itself and generates surface tension gradients (**Equations 1-3**).

Furthermore, the impact-turning dynamics at the quartz interface closely resemble elastic collisions, with a relationship between the uniform motion velocities before and after each turning process:

$v_{\mathrm{after}}=-e\cdot v_{\mathrm{before}}$	(19)

where *e* represents the coefficient of restitution, which remained stable within the range of 0.98-1.00 throughout the monitoring period.

Consequently, the system establishes a self-regulating feedback mechanism under continuous autonomous operation: velocity increases lead to higher motion frequency, which in turn amplifies energy dissipation through viscous drag and surface deformation, subsequently reducing velocity. This negative feedback loop maintains remarkably stable oscillation periods with minimal temporal fluctuation throughout the operational lifetime. The prolonged turning time observed beyond 80 minutes of operation originates from the progressive accumulation of reaction products (aluminum hydroxide precipitates and intermetallic phases) within the fuel region. This accumulation manifests in two detrimental effects: (i) increased contact line friction between the LMM and flow channel surfaces, and (ii) enhanced adhesion at the LMM-obstacle interface, both of which impede the fuel region transfer mechanism critical for efficient directional reversal.

**4.2 Platinum obstacle system: Efficient electrocatalysis**

Due to its exceptional electrocatalytic activity and electrical conductivity, the platinum obstacle system exhibits distinct behavioral characteristics, with electrochemical behavior showing pronounced evolutionary trends from the early to late stages. ^[23, 24]^ In the early stage, the rapid transition between negative and positive potential peaks reflects the rapid switching of galvanic cells:

Initial contact forms a Ga/In-Pt galvanic cell:

Anode：

$\mathrm{Ga}\longrightarrow\mathrm{Ga}^{3+}+3e^{-}$	(20)

Cathode:

$2H_{2}O+2e^{-}\longrightarrow H_{2}+2\mathrm{OH}^{-}(platinum-catalyzed)$	(21)

When the fuel region slides into contact with platinum, the Al-Pt galvanic cell becomes dominant, with the electron transfer pathway being Al-Pt-LM:

Anode：

$Al+4\mathrm{OH}^{-}\longrightarrow Al{(OH)}_{4}^{-}+3e^{-}$	(22)

Cathode:

$2H_{2}O+2e^{-}\longrightarrow H_{2}+2\mathrm{OH}^{-}(platinum-catalyzed)$	(23)

This substitutive advantage is primarily attributed to electrochemical driving force, kinetic advantages, and surface area effects. The standard electrode potential for Al^3+^/Al (-1.66 V vs. SHE) is significantly more negative than Ga^3+^/Ga (-0.53 V vs. SHE), providing a larger cell potential and greater driving force for electron transfer. ^[25]^ The oxidation of Al in alkaline environments proceeds with lower activation energy than Ga oxidation, particularly in the presence of OH^-^ ions that facilitate the formation of aluminate species. When the fuel region contacts the platinum obstacle, the effective interfacial area for the Al-Pt redox couple increases substantially (**Figure S4**), enhancing the total current flow through this pathway.

The electrode kinetics at both the Al anode and Pt cathode can be described by the Butler-Volmer equation: ^[26]^

$i=i_{0}[\exp\left( \frac{\alpha nF\eta}{RT} \right)-\exp\left( -\frac{(1-\alpha)nF\eta}{RT} \right)]$	(24)

where $i$ is the current density, $i_{0}$is the exchange current density, *α* is transfer coefficients, *n* is the number of electrons transferred, *F* is the Faraday constant, *η* is the overpotential, *R* is the gas constant, *T* is the absolute temperature

The platinum wire with high catalytic activity leads to a significant increase in the exchange current density $i_{0}$ for the hydrogen evolution reaction. According to Marcus theory: ^[27]^

$k_{ET}\propto\exp\left( -\frac{\Delta G}{RT} \right)$	(25)

where $k_{\mathrm{ET}}$ is electron transfer rate constant, $\Delta G$ is the activation free energy.

This catalytic advantage significantly reduces the activation overpotential required for the cathode reaction, accelerating the operation speed of the galvanic cell and substantially enhancing the kinetic velocity near the fuel region, thereby generating stronger surface tension gradients. This enhanced catalytic activity leads to accelerated fuel consumption rates, consequently resulting in consistently lower LMM motion velocities in platinum obstacle systems compared to quartz obstacle systems (**Figure 4c**).

In the late stage, we observe a significant decrease in potential peak values, indicating reduced electrochemical activity due to diminished aluminum activity, which resulted in decreased electron transfer rates. Meanwhile, the prolonged time for potential recovery to baseline is attributed to enhanced capacitive effects caused by the increased fuel region area. Here, we model the electrochemical system as an equivalent RC circuit, where the increased time constant $\tau= RC$ leads to slow potential recovery, consistent with exponential decay:

$v\left( t \right)=v_{0}exp(-\frac{t}{\tau})$	(26)

Furthermore, the catalytic capability of the platinum electrode decreases in later stages due to hydroxide ion adsorption, leading to rapid changes in motion periods and velocity. ^[28, 29]^ According to the Langmuir adsorption model, increasing OH⁻ surface coverage reduces available active sites for hydrogen evolution, thereby diminishing electrochemical driving force and contributing to the observed behavioral transitions. ^[30]^

**4.3 Graphite obstacle system: Complex adsorption-electrochemical coupling**

The graphite obstacle system exhibits the most complex behavior, characterized by extended turning times and significant motion period fluctuations. In the early stage, the graphite obstacle system shows collisions at the end with no directional changes detected, subsequently stabilizing at low values (<10 mV). When the fuel region is distant from the end graphite rod, the contact between liquid metal and graphite dominates the overall electrochemical behavior, generating positive potentials that initially exhibit high peak values (>60 mV) before stabilizing at lower values (<10 mV):

Anode (LM)：

$\mathrm{Ga}\longrightarrow\mathrm{Ga}^{3+}+3e^{-}$	(27)

Cathode (graphite):

$2H_{2}O+2e^{-}\longrightarrow H_{2}+2\mathrm{OH}^{-}$	(28)

The electrocatalytic activity of graphite for the hydrogen evolution reaction is significantly lower than that of platinum, with exchange current density typically ranging from 10^-6^ to 10^-5^ A∙cm^-2^. ^[31]^ For the platinum cathode, the high catalytic activity results in a large exchange current density ($i_{0}$) for hydrogen evolution, typically 10^-3^ to 10^-1^ A∙cm^-2^. ^[32]^ ^[33]^ According to the B-V equation, this lower $i_{0}$ value necessitates higher overpotentials to achieve comparable reaction rates, explaining the higher potential peaks observed with graphite electrodes.

The high initial potential peaks can be attributed to the enhanced charge separation at the graphite surface and the large effective surface area provided by graphite's porous microstructure **(Figure 5e)**, consistent with double-layer capacitance theory:

$C = \varepsilon A\cdot d^{-1}$	(29)

where $\varepsilon$ is the permittivity of the dielectric material, increased surface area *A* results in increased capacitance *C*.

Furthermore, the adsorption-desorption processes on the graphite surface significantly influence reaction kinetics. The mutual conversion between kinetic energy, chemical energy, and adsorption energy creates a complex kinetic environment, leading to extended turning times and periodic fluctuations.

In the late stage, the negative potential peaks that occur during contact between the LMM and end barriers indicate direct electron transfer at the LM-C interface. Since the hydrogen evolution reaction alters the surface morphology of graphite and introduces defect sites on its surface, the influence of graphite on the electrochemical reactions of the LMM diminishes, resulting in a significantly reduced proportion of end turning time compared to the early stages. (**Figure 4d**).^[31, 34]^ Meanwhile, the positive peaks during non-contact periods suggest the presence of long-range electrochemical effects. This may be due to electrochemical oscillation phenomena and changes in the electrochemical properties of the graphite surface caused by adsorbates.

In summary, a comprehensive analysis of the effects of different confining obstacle materials on LMM behavior reveals that the three confining obstacle systems exhibit distinctly different energy coupling modes and conversion pathways. In the quartz obstacle system, due to its electrical insulation properties, the system exhibits weak unidirectional coupling characteristics, with energy conversion following a relatively straightforward pathway: chemical energy is first converted to surface energy, and then directly transformed into the kinetic energy of the LMM. This single pathway makes the system's behavior relatively predictable. In contrast, the platinum obstacle system exhibits strong bidirectional coupling characteristics, with a more complex energy conversion pathway: chemical energy is first converted to electrochemical energy, subsequently transformed into surface energy, affecting surface tension distribution, and finally converted to kinetic energy. This bidirectional coupling mechanism makes the platinum obstacle system more sensitive to initial conditions while demonstrating richer dynamic behaviors. The graphite obstacle system presents the most complex multi-directional coupling characteristics, with an energy conversion network containing multiple intermediate steps: chemical energy is first converted to electrochemical energy, then partially transformed into adsorption energy, subsequently converted to surface energy, then transformed into kinetic energy, and finally partially dissipated as thermal energy through mechanisms such as interlayer sliding. These complex transfer processes cause the hesitation time of LMM at the ends of the graphite system to be significantly longer than that of the other two materials. This paper reveals how the physicochemical properties of obstacle materials fundamentally regulate the dynamic behavior of LMMs through multi-field coupling mechanisms, providing theoretical foundations for designing controllable LMMs.

**Supplemental Note 5: Enhanced heat transfer mechanism of LMM dynamic behavior**

To understand the temperature change mechanism of LMMs, we conduct a detailed analysis of end temperature evolution processes (**Figure S5**). During system operation, end temperature ranges exhibited significant periodic variation patterns. When LMMs approach the cold end, they transport thermal energy from the hot zone, causing localized heating phenomena. During end impact-rebound processes, fuel region position changes altered the surrounding fluid flow patterns. This created a two-stage mixing process: initially, relatively high-temperature solution from the channel interior flowed into the cold zone, followed by external cold zone solution flowing into the channel through gaps between LMMs and channel walls. These asymmetric flow patterns generated vortices that caused rapid temperature decreases in the cold end region. The cyclical process of localized heating followed by thermal recovery significantly enhanced overall heat transfer efficiency. The unique dynamic characteristics of LMMs at both terminals provide advantages not only over static systems but also demonstrate enhanced heat transfer effects compared to simple unidirectional oscillating systems. This approach shows potential for extension to more complex channel geometries, leveraging the excellent deformability and sustained motion capabilities of LMMs. ^[35]^

**Supplemental Figure 5.** **Vortexes caused by the LMM in heat transfer systems (unit: ℃).** a. The position of the observed cold end region in the entire system; b. Temporal temperature distribution changes in the cold end region during 281-292 s.

**Reference**

1.	Zhang J, Yao Y, Sheng L, Liu J, "Self-fueled biomimetic liquid metal mollusk," *Advanced Materials* 27, no. 16 (2015): 2648-55. https://doi.org/10.1002/adma.201405438.

2.	Nicholas MG, Old CF, "Liquid metal embrittlement," *Journal of Materials Science* 14, no. 1 (1979): 1-18. https://doi.org/10.1007/BF01028323.

3.	Joseph B, Picat M, Barbier F, "Liquid metal embrittlement: A state-of-the-art appraisal," *The European Physical Journal - Applied Physics* 5, no. 1 (1999): 19-31. https://doi.org/10.1051/epjap:1999108

4.	Shen M, Li Y, Hu C, et al., "The interfacial structure underpinning the Al-Ga liquid metal embrittlement: disorder vs. order gradients," *Scripta Materialia* 204, no. (2021): 114149. https://doi.org/10.1016/j.scriptamat.2021.114149.

5.	Emregül KC, Aksüt AA, "The behavior of aluminum in alkaline media," *Corrosion Science* 42, no. 12 (2000): 2051-67. https://doi.org/10.1016/S0010-938X(00)00055-X.

6.	Bagchi P, Balachandar S, "Effect of free rotation on the motion of a solid sphere in linear shear flow at moderate Re," *Physics of Fluids* 14, no. 8 (2002): 2719-37. https://doi.org/10.1063/1.1487378.

7.	John D. Anderson J, Fundamentals of Aerodynamics: McGraw hill; 2011.

8.	Magnaudet J, Mougin G, "Wake instability of a fixed spheroidal bubble," *Journal of Fluid Mechanics* 572, no. (2007): 311-37, Epub 2007/01/23. https://doi.org/10.1017/S0022112006003442.

9.	Nail SL, White JL, Hem SL, "Structure of aluminum hydroxide gel I: Initial precipitate," *Journal of Pharmaceutical Sciences* 65, no. 8 (1976): 1188-91. https://doi.org/10.1002/jps.2600650814.

10.	Snizhko LO, Yerokhin AL, Pilkington A, et al., "Anodic processes in plasma electrolytic oxidation of aluminium in alkaline solutions," *Electrochimica Acta* 49, no. 13 (2004): 2085-95. https://doi.org/10.1016/j.electacta.2003.11.027.

11.	Tanada S, Kabayama M, Kawasaki N, et al., "Removal of phosphate by aluminum oxide hydroxide," *Journal of Colloid and Interface Science* 257, no. 1 (2003): 135-40. https://doi.org/10.1016/S0021-9797(02)00008-5.

12.	Song KC, Chung IJ, "Rheological properties of aluminum hydroxide sols during sol-gel transition," *Journal of Non-Crystalline Solids* 107, no. 2 (1989): 193-8. https://doi.org/10.1016/0022-3093(89)90462-6.

13.	Jambon-Puillet E, Testa A, Lorenz C, et al., "Phase-separated droplets swim to their dissolution," *Nature Communications* 15, no. 1 (2024): 3919. https://doi.org/10.1038/s41467-024-47889-y.

14.	Liu S, Sweatman K, McDonald S, Nogita K, "Ga-based alloys in microelectronic interconnects: A review," *Materials* 11, no. 8 (2018): 1384. https://doi.org/10.3390/ma11081384, PubMed PMID: doi:10.3390/ma11081384.

15.	Liu T, Sen P, Kim CJ, "Characterization of nontoxic liquid-metal alloy galinstan for applications in microdevices," *Journal of Microelectromechanical Systems* 21, no. 2 (2012): 443-50. http://doi.org/10.1109/JMEMS.2011.2174421.

16.	Chen S, Yang X, Cui Y, Liu J, "Self-growing and serpentine locomotion of liquid metal induced by copper ions," *ACS Applied Materials & Interfaces* 10, no. 27 (2018): 22889-95. https://doi.org/10.1021/acsami.8b07649.

17.	Sun Y, Xu S, Tan S, Liu J, "Multiple electrohydrodynamic effects on the morphology and running behavior of tiny liquid metal motors," *Micromachines* 9, no. 4 (2018): 192. https://doi.org/10.3390/mi9040192 PubMed PMID: doi:10.3390/mi9040192.

18.	Zhang J, Guo R, Liu J, "Self-propelled liquid metal motors steered by a magnetic or electrical field for drug delivery," *Journal of Materials Chemistry B* 4, no. 32 (2016): 5349-57. https://doi.org/10.1039/C6TB00996D.

19.	McSkimin HJ, Andreatch P, Jr., Thurston RN, "Elastic moduli of quartz versus hydrostatic pressure at 25° and -195.8°C," *Journal of Applied Physics* 36, no. 5 (1965): 1624-32. https://doi.org/10.1063/1.1703099.

20.	Parks GA, "Surface and interfacial free energies of quartz," *Journal of Geophysical Research: Solid Earth* 89, no. B6 (1984): 3997-4008. https://doi.org/10.1029/JB089iB06p03997.

21.	Haynes WM, CRC Handbook of Chemistry and Physics: CRC press; 2016.

22.	Abrahamson J, "The surface energies of graphite," *Carbon* 11, no. 4 (1973): 337-62. https://doi.org/10.1016/0008-6223(73)90075-4.

23.	Cheng N, Stambula S, Wang D, et al., "Platinum single-atom and cluster catalysis of the hydrogen evolution reaction," *Nature Communications* 7, no. 1 (2016): 13638. https://doi.org/10.1038/ncomms13638.

24.	Shi Y, Ma Z-R, Xiao Y-Y, et al., "Electronic metal–support interaction modulates single-atom platinum catalysis for hydrogen evolution reaction," *Nature Communications* 12, no. 1 (2021): 3021. https://doi.org/10.1038/s41467-021-23306-6.

25.	Bard AJ, Standard potentials in aqueous solution. 1st Edition ed: Routledge; 1985.

26.	Dickinson EJF, Wain AJ, "The Butler-Volmer equation in electrochemical theory: Origins, value, and practical application," *Journal of Electroanalytical Chemistry* 872, no. (2020): 114145. https://doi.org/10.1016/j.jelechem.2020.114145.

27.	Marcus RA, "On the theory of oxidation‐reduction reactions involving electron transfer. I," *The Journal of Chemical Physics* 24, no. 5 (1956): 966-78. https://doi.org/10.1063/1.1742723.

28.	McCrum IT, Koper MTM, "The role of adsorbed hydroxide in hydrogen evolution reaction kinetics on modified platinum," *Nature Energy* 5, no. 11 (2020): 891-9. https://doi.org/10.1038/s41560-020-00710-8.

29.	Stamenkovic VR, Mun BS, Arenz M, et al., "Trends in electrocatalysis on extended and nanoscale Pt-bimetallic alloy surfaces," *Nature Materials* 6, no. 3 (2007): 241-7. https://doi.org/10.1038/nmat1840.

30.	Swenson H, Stadie NP, "Langmuir’s theory of adsorption: A centennial review," *Langmuir* 35, no. 16 (2019): 5409-26. https://doi.org/10.1021/acs.langmuir.9b00154.

31.	Chen F, Liu J, Chen H, Yan C, "Study on hydrogen evolution reaction at a graphite electrode in the all-vanadium redox flow battery," *International Journal of Electrochemical Science* 7, no. 4 (2012): 3750-64. https://doi.org/10.1016/S1452-3981(23)13994-0.

32.	Tetteh EB, Kim M, Savan A, et al., "Reassessing the intrinsic hydrogen evolution reaction activity of platinum using scanning electrochemical cell microscopy," *Cell Reports Physical Science* 4, no. 12 (2023): 101680. https://doi.org/10.1016/j.xcrp.2023.101680.

33.	Neyerlin KC, Gu W, Jorne J, Gasteiger HA, "Study of the exchange current density for the hydrogen oxidation and evolution reactions," *Journal of The Electrochemical Society* 154, no. 7 (2007): B631. https://doi.org/10.1149/1.2733987.

34.	Di Blasi A, Di Blasi O, Briguglio N, et al., "Investigation of several graphite-based electrodes for vanadium redox flow cell," *Journal of Power Sources* 227, no. (2013): 15-23. https://doi.org/10.1016/j.jpowsour.2012.10.098.

35.	Lee S, Mudawar I, "Enhanced model for annular flow in micro-channel heat sinks, including effects of droplet entrainment/deposition and core turbulence," *International Journal of Heat and Mass Transfer* 133, no. (2019): 510-30. https://doi.org/10.1016/j.ijheatmasstransfer.2018.12.074.
